# A preliminary study of IgG4 expression and its prognostic significance in oral squamous cell carcinoma

**DOI:** 10.1186/s12885-024-12048-5

**Published:** 2024-03-04

**Authors:** Hironobu Fukuda, Takeshi Uehara, Tomoyuki Nakajima, Mai Iwaya, Shiho Asaka, Hiroshi Kurita

**Affiliations:** 1 Department of Dentistry and Oral Surgery, Shinshu University School of Medicine, Matsumoto, Japan; 2 Department of Laboratory Medicine, Shinshu University School of Medicine, 3-1-1 Asahi, 390-8621 Matsumoto, Nagano, Japan; 3 Department of Laboratory Medicine, Nagano Children’s Hospital, Azumino, Japan

**Keywords:** IgG4, Tongue squamous cell carcinoma, Favorable prognostic factor

## Abstract

**Background:**

IgG4, which plays a pivotal role in the progression of phenotypically diverse tumors, serves as a prognostic marker because of its influence on cancer immunity. Nevertheless, the functions of IgG4 in tongue squamous cell carcinoma (TSCC) remained to be identified.

**Methods:**

To evaluate the significance of IgG4 expression in TSCC, we performed immunohistochemical analysis of patients with TSCC (*n* = 50) to evaluate the correlation of IgG4 expression with patients’ clinicopathological features and prognoses.

**Results:**

Higher IgG4 expression detected in TSCC tissues was associated with the less advanced mode of invasion (Yamamoto-Kohama [YK] 1–3) (*P* = 0.031) and with well-differentiated TSCC (*P* = 0.077). Kaplan–Meier analyses revealed that the higher IgG4 expression group exhibited better prognosis indicated by overall survival (OS) (*P* = 0.04) and recurrence-free survival (RFS) (*P* = 0.016). Univariate analysis of OS indicated that IgG4 expression was associated with longer OS (*P* = 0.061), and multivariate analysis of RFS revealed that IgG4 expression served as an independent prognostic factor for longer RFS (*P* = 0.005).

**Conclusion:**

These results indicate that relatively higher IgG4 levels serve as a favorable prognostic factor for TSCC.

## Background

Oral squamous cell carcinoma (OSCC) is the most common malignancy arising in the mouth and oropharynx [1]. Tongue squamous cell carcinoma (TSCC) is one of the most common primary sites of OSCC [2], while the frequencies of subsites of OSCC depend on race, geographical region, and lifestyle. Surgical resection is the standard treatment for TSCC. In some cases, chemotherapy and radiotherapy are performed to improve prognosis, whereas some individuals experience recurrence and metastasis after initial therapy. Therefore, the determination prognosis is a concern for surgeons. Although some reports investigated cancer biomarkers [3, 4], to our knowledge, there are no clinical predictive biomarkers.

The infiltration of tumors by immune cells serves as a prognostic factor of survival. For example, a greater lymphocytic reaction (LR) correlates with the longer survival of patients with colorectal cancer [5]. Thus, the tumor microenvironment is a factor of great interest to basic and clinical investigators. Thus, pathohistological analyses often detect inflammatory cell infiltration at the invasive front of OSCC. Despite investigations of the LR and pattern of the invasive front [6], our knowledge of the prognostic value of LR is insufficient.

Investigations of the levels of IgG4 in tumors such as esophageal cancer [7], gastric cancer [8], pancreatic cancer [9], lung cancer [10, 11], and extrahepatic cholangiocarcinoma [12] reveal that prognosis differs depending on the site of occurrence and histological type. Although progress has been made in understanding the roles of IgG4 and tumor immunity in specific tumors, certain aspects remain to be defined. Furthermore, there are no reports, to our knowledge, about the relationship between IgG4 expression and prognosis of oral cancer. Therefore, we aimed here to analyze the correlation of IgG4 expression with clinicopathological features and the prognosis of patients with TSCC.

## Methods

### Patients and materials

We enrolled 50 patients with TSCC who underwent surgical resection between January 2013 and May 2020 at Shinshu University Hospital (Matsumoto, Japan). Cases including neoadjuvant chemotherapy were excluded prior to subject selection. Two pathologists (T.U. and M.I.) reviewed the glass slides of sections of all specimens to confirm their pathological features. To evaluate the tumor stage and collect pathological features, we used the 8th UICC classification and the General Rules for Clinical and Pathological Studies on Oral Cancer. Additional data on clinical characteristics were obtained through medical records. The clinicopathological data included age, sex, pathological T stage, cervical lymph node metastasis, TNM stage, tumor size (greatest dimension), depth of invasion (DOI), histological differentiation, mode of invasion classified according to Yamamoto et al. [13] (YK classification), visual type of proliferation (superficial, exophytic, or endophytic), and venous-perineural-lymph duct invasion of the TSCC. Overall survival (OS) was defined as the interval between the data for surgical resection and those for the latest follow-up or death. Recurrence-free survival (RFS) was defined as the interval between the date of surgical resection and the date of the latest follow-up, detection of regional recurrence, or metastasis.

The Ethics Committee of the Shinshu University School of Medicine approved the protocol of the present study (Approval number: 5171).

### Histopathology, immunohistochemistry (IHC), and immunofluorescence (IF)

We prepared formalin-fixed paraffin-embedded tissue from all specimens. The representative areas of the invasive fronts of TSCC were selected in advance from hematoxylin-eosin (HE)-stained specimens. The blocks containing the invasive front of the tumor were removed using thin-walled 3 mm stainless steel needles (Azumaya Medical Instruments Inc., Tokyo, Japan). The cores were embedded in new paraffin blocks and sliced into 4 μm thick sections.

IHC to detect IgG4 was performed as follows: sections were deparaffinized in xylene, endogenous peroxidase activity was inhibited, and the sections were subsequently incubated in methanol containing 0.3% H_2_O_2_ at room temperature for 30 min. For antigen retrieval, the sections were treated at 37 °C for 20 min with 0.2% trypsin (BD Bioscience, San Jose, CA, USA) in Tris-HCl buffer (pH 7.6) containing 0.1% CaCl_2_. Sections were subsequently soaked in Tris-buffered saline containing 1% bovine serum albumin for blocking nonspecific reactions. The primary antibody used was anti-IgG4 (dilution 1:50, The Binding Site, Birmingham). The sections were incubated with the primary antibody for 1 h at room temperature. To visualize immune complexes using IHC, the sections were immersed in DAB solution, and the samples were counterstained with hematoxylin. FOXP3 was autostained using the Bond-III system (Leica, Wetzlar, Germany), and BOND Epitope Retrieval Solution 2 was utilized for antigen retrieval. The primary antibody used was anti-FOXP3 (dilution 1:100, Clone 236 A/E7; Abcam).

IF detection of IgG4 was performed using a secondary antibody labeled with Alexa Fluor 647 (Invitrogen, Carlsbad, CA, USA) for 45 min at room temperature. Microscopic analysis was conducted using an Axio Imager Z2 (Zeiss, Jena, Germany). Images were captured using an Isis FISH imaging system (Metasystems, Altlussheim, Germany).

### Evaluation of IHC data

To evaluate IgG4 and Foxp3 expression, IgG4-positive plasma cells and Foxp3-positive cells in the tumor stroma of each case were analyzed. Areas with the highest density of the cells were selected and directly measured using a light microscope at each location (40× eyepiece). The field number of the eyepiece = 26.5, the field diameter = 0.6625 mm, and the field area of the high magnification = 0.345 mm^2^.

Clinicopathological analysis was performed by dividing the median values of IgG4-positive plasma cell counts into the high IgG4 and low IgG4 expression groups. Prognostic analysis was conducted by categorizing Foxp3-positive cell counts into high Foxp3 and low Foxp3 expression groups based on their median values.

### Statistical analysis

The chi-squared test was applied to assess the statistical significance of differences. The Kaplan–Meier method was used to estimate OS and RFS rates, and the log-rank test was used to compare differences in OS and RFS rates between groups. The Cox proportional hazard regression model was used to conduct univariate and multivariate analyses. Variables with *P* < 0.05 in univariate analyses were included in the multivariate analyses. Receiver operating characteristic (ROC) curve analysis was conducted to evaluate the diagnostic performance of IgG4 expression associated with OS and RFS. Cut-off values were determined according to the Youden’s index.

*P* < 0.05 represents a significant difference. Data were compiled and analyzed using IBM SPSS Statistics 27.0.

## Results

### IgG4 expression and clinicopathological features

**Fig. 1:**
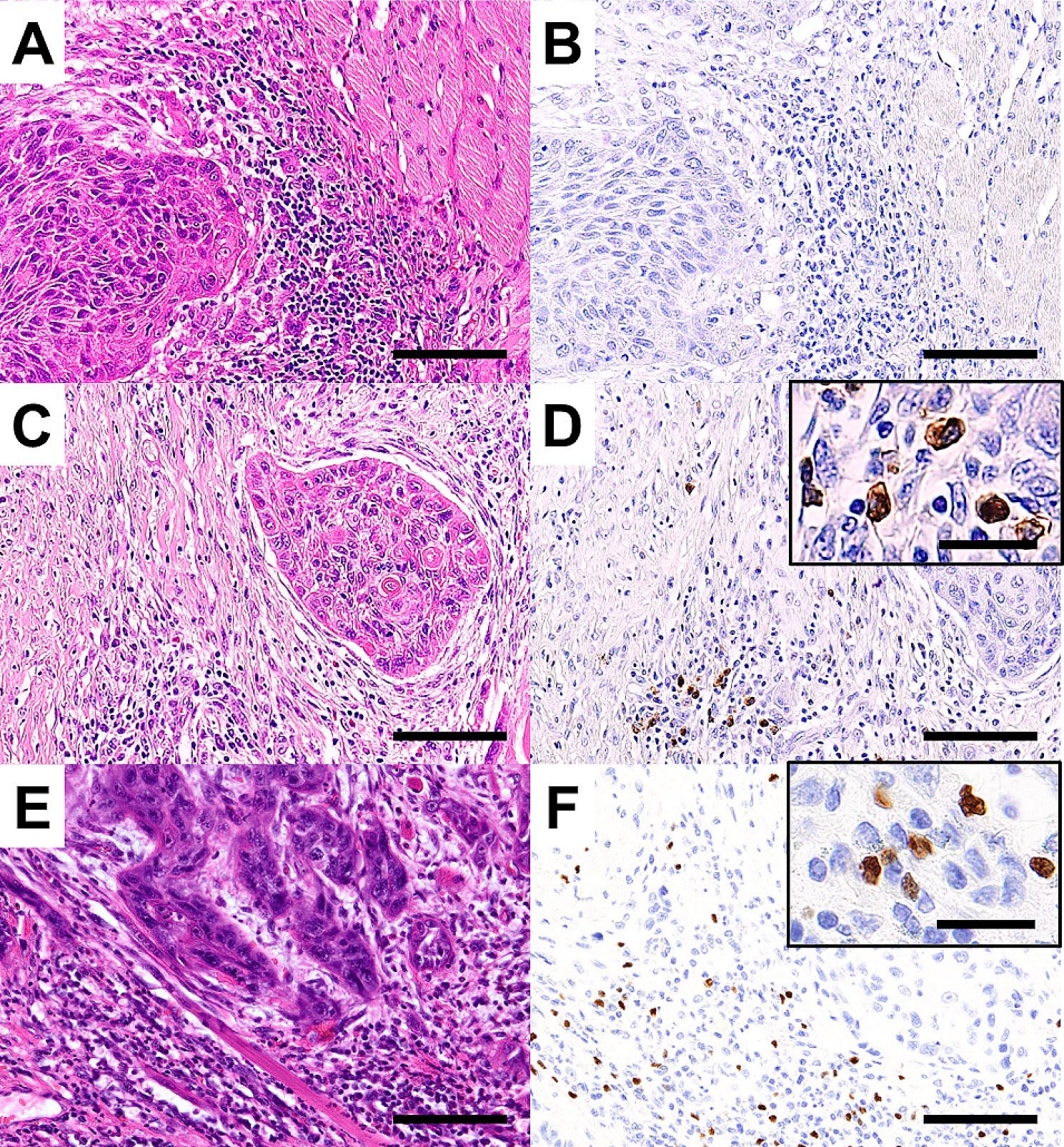
Representative images of IgG4 expression. Representative features of hematoxylin-eosin (HE)-stained tissues (**A**) and immunochemical analysis of low IgG4 expression (**B**). Representative features of HE-stained tissues (**C**) and immunochemical analysis of high IgG4 expression (**D**). Representative features of HE-stained tissues (**E**) and immunochemical analysis of high Foxp3 expression (**F**). Bar indicates 100 μm (magnified panel = 25 μm)

Inflammatory cell infiltration and fibrosis were identified around the cancer tissues. These inflammatory cells comprised mainly lymphocytes and plasma cells (Fig. 1A and C). IHC analysis revealed an uncharacteristic appearance of the pattern of IgG4-positive plasma cells. There were various cases, such those in which almost no IgG4-positive plasma cells were observed (Fig. 1B), cases in which IgG4-positive plasma cells were sparse, and cases characterized by aggregation (Fig. 1D). Foxp3-positive cells were observed around the invasive front of the tumor (Fig. 1E and F).

**Fig. 2:**
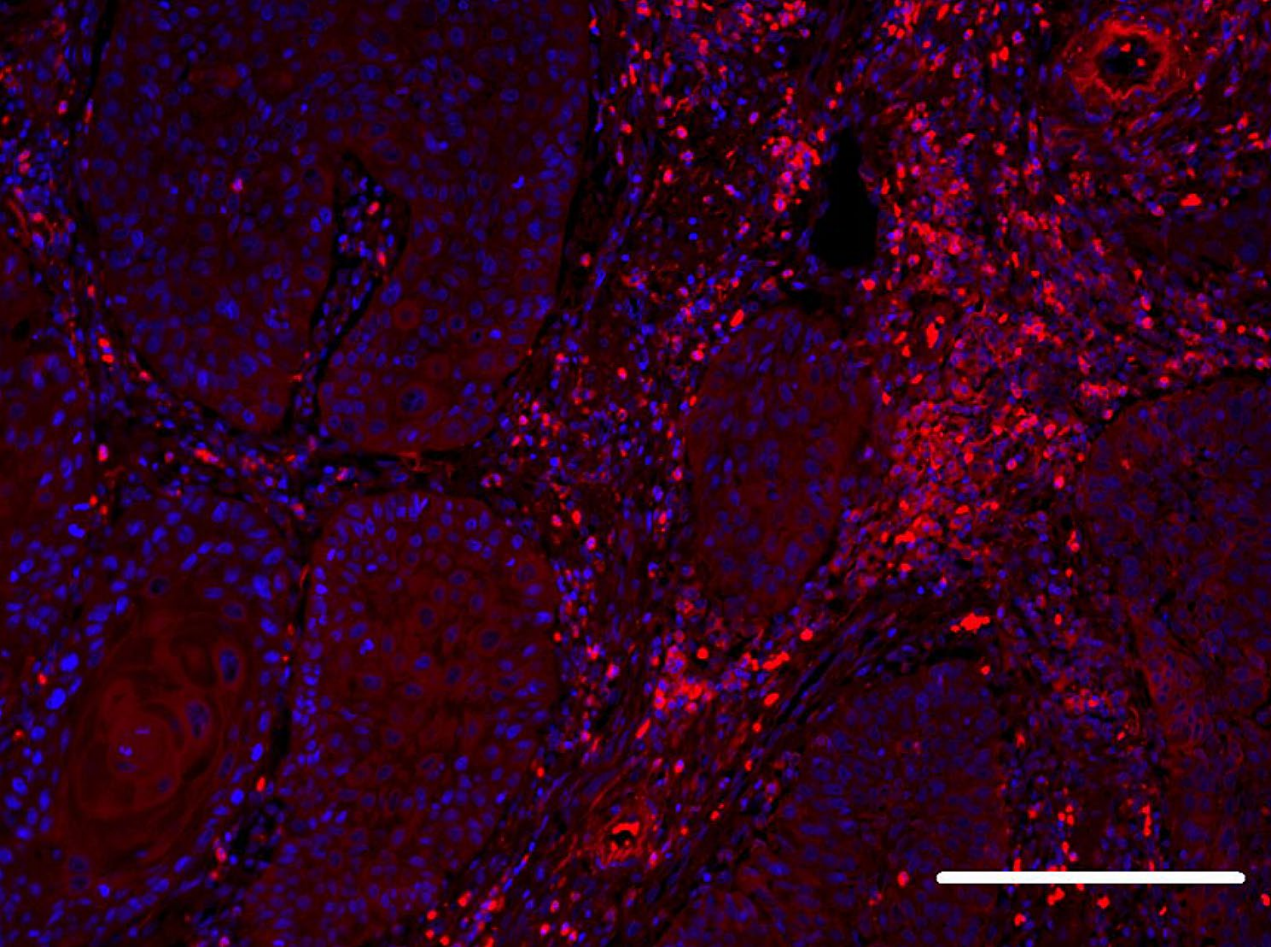
Immunofluorescence detection of plasma cells. IgG4-positive plasma cells were diffusely distributed in the tumor stroma and near the tumor. Immunofluorescence was used to detect IgG4 (red). Nuclei were visualized using DAPI. Bar indicates 200 μm

Furthermore, in some cases, IF analysis detected IgG4-positive plasma cells diffusely distributed in the tumor stroma and around the tumor. (Fig. 2).

Table 1 shows the correlation between IgG4 expression and clinicopathological characteristics of patients with TSCC. The mode of invasion (YK classification) significantly correlated with IgG4 expression (*P* = 0.031). Higher IgG4 expression was detected in the less advanced modes of invasion (YK1-3). Furthermore, well-differentiated TSCC was associated with higher IgG4 expression (*P* = 0.077), although the differences were not statistically significant.

**Table 1:** Analyses of IgG4 expression and clinicopathological features in TSCC

Variables		IgG4 expression		
		High	Low	
		(*n* = 25)	(*n* = 25)	*P* value
Age	≤ 67	13	11	0.571
	> 67	12	14	
Sex	male	15	14	0.774
	female	10	11	
Pathological T stage	T1/2	18	15	0.37
	T3/4a	7	10	
Metastasis of cervical lymph node	Absent	17	16	0.765
	Present	8	9	
TNM stage	I-II	14	11	0.396
	III-IV	11	14	
Tumor size (mm)	≤ 25 mm	15	11	0.258
	> 25 mm	10	14	
Depth of Invasion (mm)	≤ 8 mm	13	12	0.777
	> 8 mm	12	13	
Histological differentiation	Well	19	13	0.077
	Mod/Poor	6	12	
Mode of invasion (YK classification)	1–3	21	14	**0.031**
	4 C/4D	4	11	
Visual type of proliferation	Endophytic	17	16	0.765
	Superficial/Exophytic	8	9	
Venous invasion	Positive	10	11	0.774
	Negative	15	14	
Perineural invasion	Positive	9	13	0.254
	Negative	16	12	
Lymph duct invasion	Positive	6	4	0.48
	Negative	19	21	
Foxp3 expression	High	13	12	0.777
	Low	12	13	

In contrast, there were no significant differences between IgG4 expression, and the variables as follows: age, sex, pathological T stage, metastasis of the cervical lymph node, TNM stage, tumor size, DOI, visual type of proliferation, venous-perineural-lymph duct invasion, and Foxp3 expression.

### IgG4 and Foxp3 expression as a prognostic factor for favorable OS and RFS

**Fig. 3:**
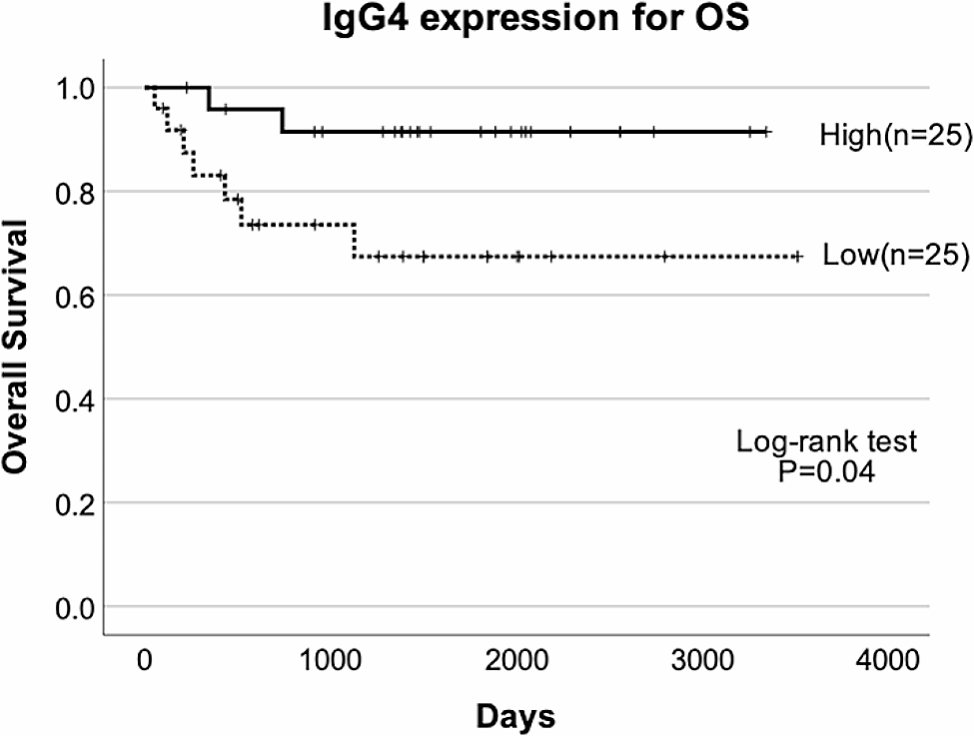
Kaplan–Meier analysis of the prognostic value of IgG4 expression associated with OS. High IgG4 expression levels were associated with favorable OS (log-rank test *P* = 0.04)

**Fig. 4:**
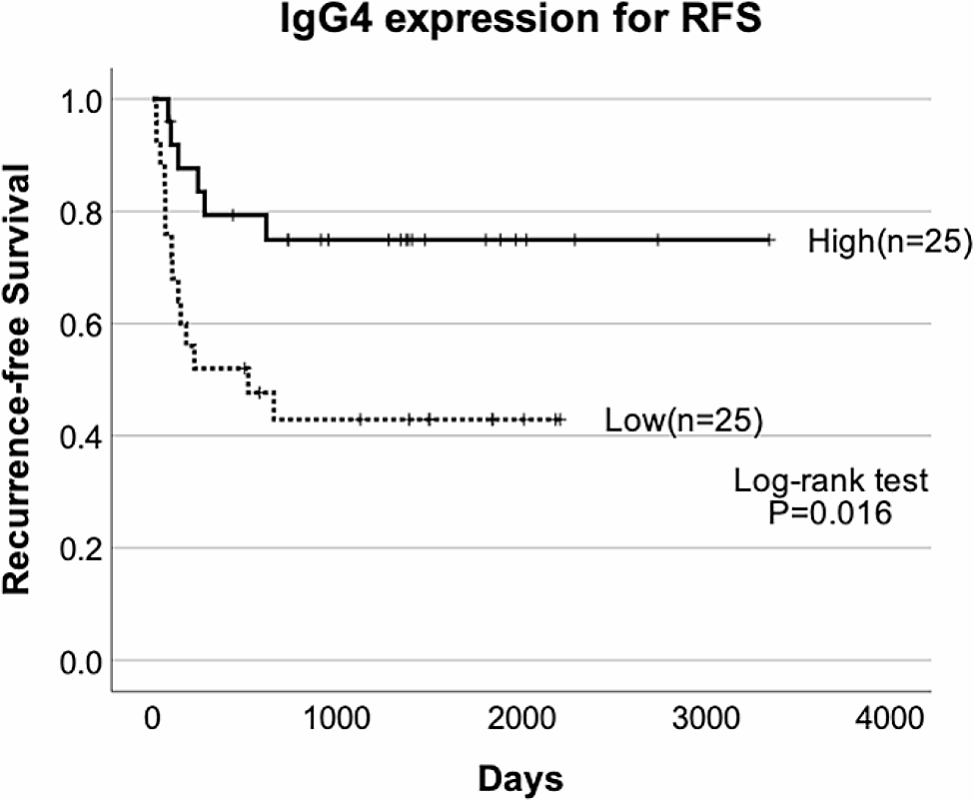
Kaplan–Meier analysis of the prognostic value of RFS associated with IgG4 expression. A high IgG4 expression level was associated with favorable RFS (log-rank test *P* = 0.016)

Better OS and RFS rates were significantly associated with the higher IgG4 expression group (OS, *P* = 0.04; RFS, *P* = 0.016, log-rank test) (Figs. 3 and 4). These results indicated that infiltration of IgG4-positive plasma cell was related to better prognosis. Additionally, Foxp3 expression was significantly associated with better OS and RFS (OS, *P* = 0.035; RFS, *P* = 0.012, log-rank test).

Table 2 shows univariate analyses of prognostic factors for OS. Univariate analysis revealed that metastasis of the cervical lymph node (*P* = 0.011) and TNM stage (*P* = 0.045) were significantly associated with OS; and IgG4 and Foxp3 expression tended to be associated with OS, although the differences were not statistically significant (*P* = 0.061, 0.055, respectively).

**Table 2:** Univariate analyses of prognostic factors for overall survival of patients with TSCC

Variables	Univariate analysis		
	HR	(95% CI)	*P* value
Age: ≤67 years vs. > 67 years	1.28	(0.34–4.76)	0.715
Sex: female vs. male	0.31	(0.08–1.22)	0.094
Pathological T stage: T1/2 vs. T3/4a	2.21	(0.59–8.26)	0.24
Metastasis of cervical lymph node: Absent vs. Present	6.20	(1.53–25.14)	**0.011**
TNM stage: I-II vs. III-IV	5.02	(1.04–24.32)	**0.045**
Tumor size(mm): ≤25 mm vs. > 25 mm	2.62	(0.66–10.51)	0.173
Depth of Invasion(mm): ≤8 mm vs. > 8 mm	2.70	(0.67–10.87)	0.161
Histological differentiation: Mod/Poor vs. Well	0.64	(0.17–2.40)	0.513
Mode of invasion (YK classification): 1–3 vs. 4 C/4D	2.24	(0.60–8.34)	0.231
Visual Type of proliferation: Superficial/Exophytic vs. Endophytic	0.26	(0.06–1.03)	0.055
Venous invasion: Negative vs. Positive	1.23	(0.33–4.57)	0.761
Perineural invasion: Negative vs. Positive	3.21	(0.80-12.88)	0.099
Lymph duct invasion: Negative vs. Positive	2.50	(0.62–10.01)	0.196
IgG4 expression: Low vs. High	0.22	(0.05–1.07)	0.061
Foxp3 expression: Low vs. High	0.21	(0.04–1.04)	0.055

Further analyses of the prognostic value of IgG4 expression for RFS are shown in Table 3. Metastasis of the cervical lymph node (*P* = 0.008), perineural invasion (*P* = 0.020), IgG4 expression (*P* = 0.022), and Foxp3 (*P* = 0.018) were significant prognostic factors in univariate analyses. Furthermore, multivariate analysis revealed that metastasis of the cervical lymph node (*P* = 0.017), IgG4 expression (*P* = 0.005) and Foxp3 (*P* = 0.011) were independent factors for the recurrence of TSCC.

**Table 3:** Univariate and multivariate analyses of prognostic factors for recurrence-free survival of TSCC

Variables	Univariate analysis				Multivariate analysis		
	HR	(95% CI)	*P* value		HR	(95% CI)	*P* value
Age: ≤67 years vs. > 67 years	0.97	(0.40–2.32)	0.936				
Sex: female vs. male	0.59	(0.25–1.43)	0.242				
Pathological T stage: T1/2 vs. T3/4a	2.14	(0.89–5.17)	0.091				
Metastasis of cervical lymph node: Absent vs. Present	3.31	(1.36–8.05)	**0.008**		3.29	(1.23–8.76)	**0.017**
TNM stage: I-II vs. III-IV	2.46	(0.98–6.19)	0.056				
Tumor size(mm): ≤25 mm vs. > 25 mm	2.07	(0.84–5.06)	0.112				
Depth of Invasion(mm): ≤8 mm vs. > 8 mm	2.33	(0.93–5.86)	0.072				
Histological differentiation: Mod/Poor vs. Well	0.51	(0.21–1.23)	0.134				
Mode of invasion (YK classification): 1–3 vs. 4 C/4D	1.79	(0.73–4.40)	0.201				
Visual type of proliferation: Superficial/Exophytic vs. Endophytic	0.93	(0.37–2.34)	0.880				
Venous invasion: Negative vs. Positive	1.73	(0.72–4.16)	0.221				
Perineural invasion: Negative vs. Positive	2.99	(1.19–7.52)	**0.020**		2.22	(0.86–5.77)	0.101
Lymph duct invasion: Negative vs. Positive	1.72	(0.63–4.74)	0.293				
IgG4 expression: Low vs. High	0.33	(0.13–0.85)	**0.022**		0.22	(0.07–0.62)	**0.005**
Foxp3 expression: Low vs. High	0.31	(0.12–0.82)	**0.018**		0.27	(0.10–0.74)	**0.011**

**Fig. 5:**
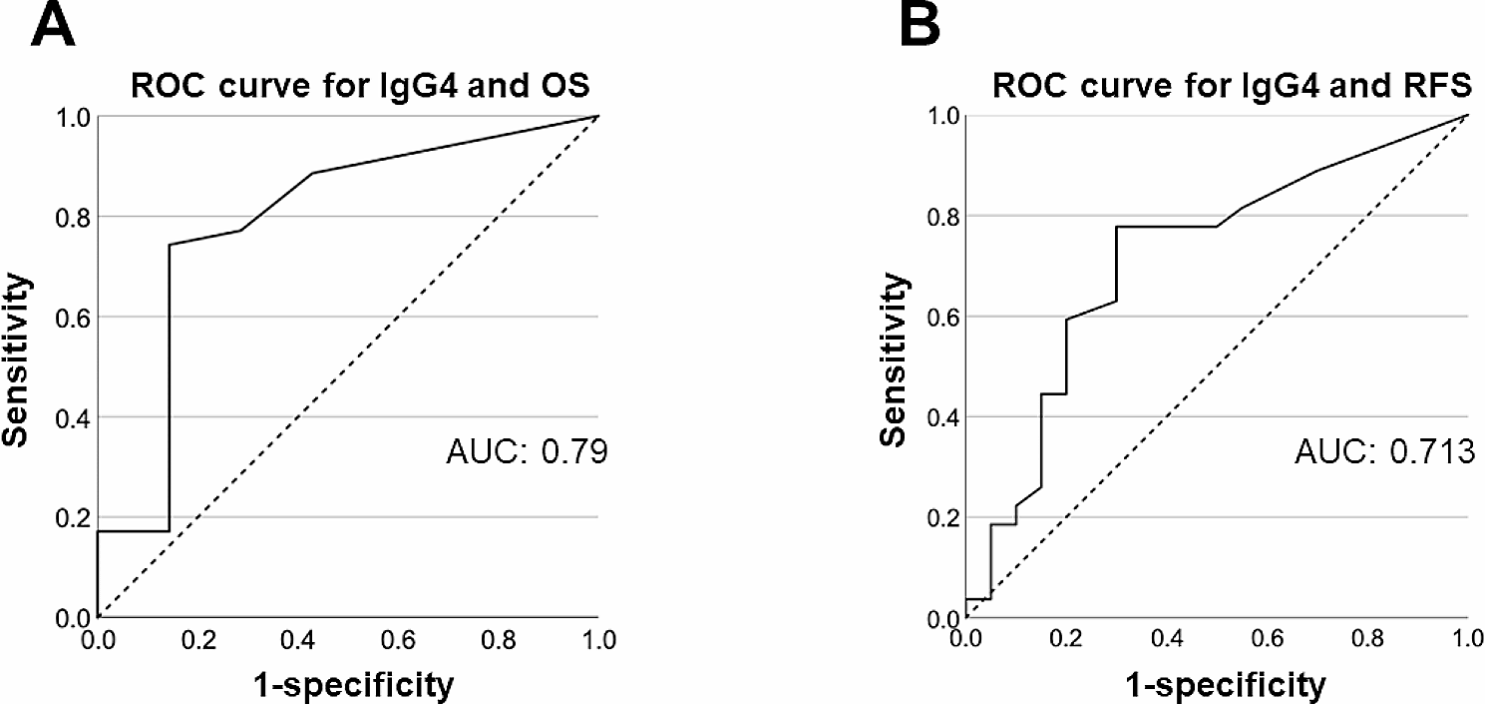
ROC curve analysis of IgG4 expression associated with OS (**A**) and RFS (**B**). The AUCs of OS and RFS showed fair discriminatory accuracy

The results of the ROC analysis of IgG4 expression for OS and RFS and the areas under the ROC curve (AUC) are shown in Fig. 5. The analysis revealed IgG4 expression helped predict OS (AUC = 0.79, sensitivity = 0.743, specificity = 0.857, cut-off value = 2.5) and RFS (AUC = 0.713, sensitivity = 0.778, specificity = 0.7, cut-off value = 3.5). AUCs of OS and RFS AUCs showed fair discriminatory accuracy.

## Discussion

Here we present the first investigation, to our knowledge, of the relationship between IgG4 expression and the prognosis of TSCC. Our present findings are consistent with reports of high IgG4 expression in the tumor stroma leading to better prognosis of lung cancers [10]. However, in gastric and pancreatic cancers, an abundance of IgG4-positive cells is associated with poor prognosis [8, 9].

Myeloid-derived suppressor cells (MDSCs) may explain the abundance of IgG4, leading to better survival. MDSCs are immature bone marrow-derived cells that increase in numbers in tumor tissue, lymph nodes, and peripheral blood of patients with cancer. Cytokines secreted by cancer cells mobilize MDSCs from the bone marrow to the tumor microenvironment, and MDSCs suppress cancer immunity by inducing Tregs and acting on CD8-positive T cells and NK cells [14–16]. MDSC populations, which are rather highly heterogeneous, can be divided into the major groups classified as myeloid MDSCs and monocytic MDSCs, distinguished by their varying degrees of differentiation.

The properties and distribution of IgG4 in the oral cavity may exert specific effects on MDSCs. For example, evidence indicates that IgG4 serves to blockade antitumor immunity [17]. However, the oral cavity is basically an IgG4-rich region, with a different environment comprising coexisting hard and soft tissues. The intraoral abundance of microorganisms and mechanical stress caused by eating and speaking causes chronic inflammation; and the severity of periodontal diseases is associated with different IgG subtypes [18]. Moreover, the numbers of IgG4-positive plasma cells tend to be higher compared with those of other regions [19]. Hence, IgG4 expression in the oral cavity differs from that in other tissues.

IgG4 molecules inefficiently cross-link antigens to form immune complexes [20]. Furthermore, IgG4 may suppress the activity of other IgG subclasses [7]. For example, abundant IgG4 levels inhibit immune complex formation and may contribute to the suppression of MDSCs [11], and IgG4 therefore may be indirectly involved in activating cancer immunity.

IgG4 expression is associated with poor prognoses of adenocarcinomas of the pancreas, liver, and gastric tissue [8, 9, 21]. Differences in the prognostic impact of IgG4 expression may be organ dependent and possibly caused by underlying differences in the immune regulation of tumors. For example, in lung cancer tumor-infiltrating lymphocytes (TILs) and cancer-associated fibroblasts (CAFs) correlate more strongly with SCC than with lung adenocarcinoma, and TILs and CAFs are associated with MDSCs [22]. These findings may represent collateral evidence for differences in IgG4 involvement with oncogenesis and tumor progression according to histological type.

The activities of B cells and humoral immunity positively correlate with the activation of the Fc-gamma receptor (FcγR), leading to carcinogenesis [23]. However, IgG4 binds Fc-receptors with low affinity [24] and therefore may play an important role in immune evasion mechanisms [7]. High IgG4 expression associates with worse prognosis of esophageal cancer. However, this analysis is not based on the log-rank test comparisons of survival curves. Indeed, IgG4 exhibits specific Fc-Fc binding properties. Thus, the identification of differences in the immune systems of vertebrates that interact with tumors require intensive investigations.

The behaviors of Tregs in oral diseases have been widely investigated in recent decades, and some of these studies may support our conclusion about the functions of Tregs in oral cancer. For example, compared with healthy subjects, Tregs are elevated in patients with head and neck squamous cell carcinoma (HNSCC) [25]. However, the significance of Tregs as a prognostic factor remains controversial, although Tregs serve as a favorable prognostic factor in HNSCC [26, 27]. Our study also showed that Treg was a favorable prognostic factor in HNSCC. Furthermore, the number of cytotoxic T cells depends on whether Tregs improve the prognosis of HNSCC [28]. In contrast, IgG4-related diseases may be associated with increased Tregs [29]. Thus, Tregs and IgG4 are often abundant in HNSCC, and the prognostic value of IgG4 may depend on the number of cytotoxic T cells in tumor tissue.

One limitation of our study is that it was retrospective, and we did not analyze serum IgG4 levels in each case. Therefore, the relationship between serum IgG4 levels and clinicopathology is a topic for future study.

Here we specifically conducted IHC analysis to assess IgG4 expression, and further studies are required to identify other factors involved in tumor immunity of TSCC. For example, analysis of the effects of IgG4 on cultured tumor cells and immune cells functionally associated with tumors may contribute to IgG4’s role in TSCC.

## Conclusion

The oral cavity represents a unique environment in which the involvement of IgG4 expression leads to a favorable prognosis and thus may represent an important stepping stone in the development of improved treatment of OSCC.

## Data Availability

Data generated and analyzed during the current study are available from the corresponding author.
